# Forced Intercourse in America: A Pandemic Update

**DOI:** 10.21203/rs.3.rs-2591896/v1

**Published:** 2023-03-01

**Authors:** William G. Axinn, Brady T. West, Heather M. Schroeder

**Affiliations:** University of Michigan–Ann Arbor; University of Michigan–Ann Arbor; University of Michigan–Ann Arbor

**Keywords:** forced intercourse, population trends, COVID-19 pandemic, intimate partner violence

## Abstract

**Background:**

Measures of forced intercourse from the U.S. National Center for Health Statistics (NCHS) indicate this a high prevalence among U.S. women that is likely to produce unintended pregnancies. However, NCHS did not measure forced intercourse during the pandemic, limiting knowledge of recent prevalence rates.

**Methods:**

We use multiple nationally-representative, cross-sectional surveys representing the U.S. population from 2011 to 2022 to document these trends. This includes measures from the National Survey of Family Growth, the Panel Study of Income Dynamics Transition into Adulthood Supplement, and the American Family Health Study (AFHS) to provide population estimates of forced intercourse.

**Results:**

Reports of forced intercourse remained high during the pandemic, with more than 25% of U.S. females over 40 reporting lifetime forced intercourse in the AFHS (number of females in AFHS: 1,042). There was a significant increase among females aged 24–28 (p<.05) and rates are highest for those who did not complete college. Among females 24–28, 32.5% (S.E. = 5.7%) with less than 4 years of college reported forced intercourse, a significantly (p<.05) higher rate than among those with higher education.

**Conclusions:**

Rates of forced intercourse among U.S. women remained high during the pandemic, increasing significantly in early adulthood. This exposure to forced intercourse is likely to produce an increase in unintended pregnancies and other sexual, reproductive, and mental health problems.

## Background

The U.S. National Center for Health Statistics (NCHS) monitors national rates of forced intercourse as an important element of reproductive health, with potential consequences for rates of unintended pregnancy and childbearing (1,2). All forms of sexual assault are important for health and wellbeing because the consequences include many negative physical and mental health outcomes. These adverse consequences of sexual assault include sexually transmitted infections, unintended pregnancies, or injuries including death (3–5). Robust associations have been reported between sexual assault and post-traumatic stress disorder (6), major depressive disorder (7), generalized anxiety disorder (8), and suicide attempt (9). The experience of forced intercourse, in particular, can have severe long-term adverse health consequences (10).

The COVID-19 pandemic, and measures taken to reduce transmission, dramatically changed daily life: work in many sectors stopped; some employees began working remotely; and childcare, meal preparation, and other daily tasks returned to the home, at least more often. These interruptions reduced social interactions, with potential consequences for courtship processes like meeting potential partners, dating, and transitioning to sexual, co-residential and marital relationships. Early evidence from the pandemic indicates that rates of intercourse in the U.S. declined overall (11).

Before the pandemic, the U.S. had a high prevalence of forced intercourse (12). For example, in 2011–2013, 25% of women reported ever experiencing forced intercourse by age 44 (Table 1). The pandemic may have decreased rates of forced intercourse by reducing sexual activity overall (eTable 1 provides more detail [see Additional file 1]). However, the pandemic also had the potential to *increase* forced intercourse. It is possible that pandemic-related changes forced couples to spend more time together, even those in unhealthy relationships. For couples who would have divorced or separated (including seeking temporary shelters from an abusive partner), the pandemic may have made moving or finding alternative housing more difficult. Such circumstances would have high potential to intensify negative relationships. Physical violence between intimate partners is considered the most severe dimension of negative relationships, reproduced through sexual violence and forced intercourse, making these among the worst forms of intimate partner violence (12,13). Forced intercourse also has clear implications for pregnancy and childbearing: forced intercourse rarely involves effective contraception, thus increasing the probability of unintended pregnancy (14).

The pandemic also interrupted education; for many young adults, this meant moving away from college campuses to continue their educations remotely or dropping out. Research demonstrates that the probability of experiencing a sexual assault is high among college students (15–18). The Association of American Universities estimates that on many campuses more than 20% of undergraduate women experience sexual assaults each year (15). Those rates remained consistently high on American university campuses just before the pandemic, actually increasing at some universities (19). However, multiple nationally-representative studies show that students enrolled in college are less likely to experience sexual assaults than peers not enrolled in college (12,20–22).

We document the extent to which rates of forced intercourse in the U.S. population remained stable before the pandemic and changed during the pandemic, including the association between educational attainment and forced intercourse. We use the NCHS’s National Survey of Family Growth (NSFG) and two other nationally representative data sources which replicate the NSFG measurement of forced intercourse: the U.S. Panel Study of Income Dynamics Transition into Adulthood Supplement (PSID-TAS, 2017 and 2019) and the American Family Health Study (AFHS 2020–2022). A unique data resource, the AFHS[1] is an important breakthrough in survey design for population science (23), and as a web survey it was ideally positioned to succeed during the pandemic. These three data sources are ideal for measuring trends, or disruptions in trends, because they use the same wording to measure forced intercourse. However, these studies only measure forced intercourse. Forced intercourse represents just under half of all sexual assaults, but it is a severe form of sexual assault, with many associated health risks and with potential for long-term adverse consequences (10,15,17).

[1] Please see the AFHS website for more information: https://afhs.isr.umich.edu/

## Methods

The U.S. has multiple studies of the general population that can be used for population-level investigation of sexual assault, including forced intercourse. The National Research Council recently conducted a thorough review of the survey measurement of sexual assault, identifying many important strengths and weaknesses.(24) For example, they concluded that the U.S. National Crime Victimization Survey (NCVS) likely underestimates the prevalence of sexual assault because of the in-person administration of NCVS interviews (which reduces privacy), the use of a screening measure with little context, and the criminal behavior focus of that survey (24). By contrast, surveys conducted in modes that offer more privacy, place questions about sexual assault in a context of other relationship and sex-related questions, and are presented to respondents as measuring a broader range of experiences than crimes are likely to obtain more complete reports of sexual assault. The three survey data sources used accomplish each of these goals: more private modes, sexual assault is asked in the context of all relationship experiences, and the surveys do not focus on crime. As fully nationally representative data sources, the three surveys used represent all Americans independent of their socioeconomic status, race, ethnicity, language, nationality, sex, gender identity, sexual orientation, religion, geography, ability, age, or culture.

The NSFG was specifically designed to serve as the national source of statistics on sexual relationships, reproductive health, childbearing, and other related topics (2). It is an important data source for studying sexual assault because it features lifetime histories of sexual partnerships, pregnancies, sex, and reproductive health issues. It also uses audio-computer assisted self-interviewing (ACASI) to provide privacy and immediate response encryption while asking questions about sensitive reproductive health issues, such as forced intercourse. The NSFG offers a nationally-representative sample of persons aged 15–49, private measurement of experiences with forced intercourse *outside of a criminal context*, and response rates that generally exceed 70% (reducing potential nonresponse bias (25)). Specific wording used in the NSFG measures and a discussion of issues related to respondent interpretation of the questions were previously published (12).

We used publicly available NSFG data (2011–2017) to update the pre-pandemic trends in experiences of forced intercourse. Professional interviewers conducted face-to-face interviews with ACASI for some sensitive items (including forced intercourse) in a probability-based, nationally-representative, area-clustered sample. The NSFG data collection from 2011–2013 interviewed 10,416 respondents aged 15–44 (72.8% response rate), from 2013–2015 interviewed 10,210 respondents aged 15–44 (69.3% response rate), and from 2015–2017 interviewed 10,094 respondents aged 15–49 (65.3% response rate) (26). NSFG measures of forced intercourse are limited to those aged 18 and over. Weights constructed to account for the complex nature of the NSFG sample design, along with stratum and cluster codes for variance estimation, were used for our analysis. Note that the NCHS chose not to release forced intercourse measures in unrestricted public data for the NSFG 2017–2019, despite having done so previously.

The 2017 PSID-TAS[2] collected data from 2,526 males and females aged 18–28 (87% response rate). The PSID-TAS age restriction necessitated restricting the ages of other samples when comparing to these data. Data were collected by telephone from October 2017 to June 2018. The 2019 PSID-TAS^2^ introduced a web option for data collection in addition to telephone, which yielded interviews from 2,595 males and females between November 2019 and July 2020 (86% response rate). Both studies replicated the NSFG wording for measures of forced intercourse. Estimates in this analysis incorporated the cross-sectional weight that accounts for the original PSID selection probability, non-response, differential eligibility, and calibration to known features of the population of interest (27,28).

The AFHS[3] was designed to collect data from two national sample replicates, fielded from April 2020 through April 2022. The AFHS uses a mixed-mode web/mail protocol to screen a random address-based probability sample of U.S. households and identify eligible household members aged 18–49. One randomly selected eligible respondent within each household is invited to complete the study, which *replicates the content of the NSFG*. Both the screening questionnaire and the survey were designed to be completed online, but can also be completed by returning paper questionnaires through the mail; like the use of ACASI to measure forced intercourse in the NSFG, the use of these self-administered modes is expected to increase reporting of this type of sensitive experience (29). Estimates in this analysis focused on 1,914 respondents aged 18–49 who responded to questions on forced intercourse and 596 respondents aged 18–28 to match the PSID-TAS. These estimates incorporated the final survey weights, in addition to bootstrap replicate weights that correctly reflect the stratified sample design and account for all nonresponse adjustments and calibration adjustments applied to the sampling weights. Particularly important, no area cluster sampling was necessary for the AFHS, introducing cost and statistical efficiency relative to face-to-face approaches (23). One consequence of this efficiency is the ability to achieve reliable estimates with fewer interviews. All analyses were performed using appropriate SURVEY procedures in SAS (version 9.4). The code is available upon request.

### Outcome Measures

Our binary indicator for ever being forced to have intercourse (1=Yes, 0=No) was derived from two questions. If the respondent indicated that first sex was not voluntary or if they reported ever being forced to have sex, they were coded as ever forced to have intercourse.[4]

### Analytic Approach

We first generated descriptive estimates of the percentages of subgroups defined by age and gender ever experiencing forced intercourse in 2011–2017, and 2020–2022 among those aged 18–49 based on the NSFG and AFHS data sources. Next, we focused on younger Americans, adding the PSID-TAS data (2017 and 2019) and restricting the age range to match PSID-TAS (18–28). Finally, we estimated the association between the cumulative experience of college attendance and rates of ever experiencing forced intercourse, focusing on men and women aged 24–28. By these ages, many Americans with college experience have finished attending college. This approach allowed us to compare the cumulative experience of ever being forced to have intercourse across different levels of completed college experience. We used a dichotomous measure of college attendance—less than four years of college attendance vs. four or more years of college attendance—to maintain comparability across data sources.

Though we provide estimates across the full age ranges studied (18–49 and 18–28), we also provide estimates for narrower age-range subgroups (e.g., 20–24). This addition is important because narrower age groups differ substantially in exposure to the risk of ever being forced to have intercourse. Not only do more years of life increase the time of exposure, but different birth cohorts also live through different periods that may alter risks. For example, new risks created by social distancing during the pandemic may differ greatly by birth cohort. Thus, even within the analyses of those aged 18–28, we provide additional documentation of even narrower age groups, 18–23 and 24–28, because these cohorts differ in their exposures to risk.

[2] Data are publicly available.

[3] Data are being processed for public release. All procedures involving human subjects for the AFHS were approved by the University of Michigan Health Sciences and Behavioral Sciences IRB (HUM00167171).

[4] For males from PSID-TAS, only the forced male-female question is asked.

## Results

### Estimated Rates of Forced Intercourse

We estimate that approximately 20% of U.S. women aged 18–49 have ever been forced to have vaginal intercourse, and that this rate appears roughly stable into the pandemic (Table 1). However, the age-Specific rates demonstrate increases during the pandemic among women aged 20 and over, with the biggest difference among women aged 20–24. This difference is a significant increase from both NSFG 2015–2017 (p<.10) and NSFG 2011–2013 (p<.05). We focused on the younger ages in subsequent analyses.

Men also had this experience, at a lower rate (Table 1). From 2011 to 2017 and into the pandemic (2020–2022), these rates appeared stable, with approximately 6% of men reporting lifetime experiences of forced intercourse (Table 1). Forced intercourse for men includes both vaginal intercourse forced by women and oral or anal intercourse forced by men.

### Ages 18–28 only

Next, we focused on ages 18–28 to add measures from the PSID-TAS and focus on pandemic-Specific increases in forced intercourse at younger ages. First, consider women (Table 2). Reports of lifetime experience of forced intercourse are lower in the PSID-TAS sample than in NSFG or AFHS. However, comparing AFHS results to other results in Table 2, we find an increase in reporting of lifetime forced intercourse among those aged 18–28 during the pandemic that is particularly large among those aged 24–28. The highest pre-pandemic estimate for this age group is the 2015–2017 NSFG and the pandemic-Specific estimate from AFHS is ten percentage points higher. This difference is significant (p<.05) (Table 2).

Although the modes of data collection varied across studies, with NSFG using ACASI, PSID-TAS 2017 using primarily phone, PSID-TAS 2019 using primarily web, and AFHS using primarily web, observed differences across data sets do not appear to correspond to mode of interview. Rather, the AFHS, which repeats all of the NSFG context of questions about lifetime experiences of all types of sexual relationships and reproductive health issues, looks the most similar to NSFG. PSID-TAS has much less content on sexual and reproductive health than either NSFG or AFHS, which may be part of the explanation for lower observed rates in that study. Although the self-administered web mode in AFHS may have produced more honest reporting of experience with forced intercourse, the vast majority of measures unlikely to be affected by the pandemic remained consistent between the NSFG and AFHS.(30) But national estimates of the prevalence of forced intercourse for women aged 24–28 from 2020 to 2022 are higher than all other time periods.

Next, consider younger men (Table 2). Three results are clear among men aged 18–28. First, similar to women, rates of reporting lifetime experience of forced intercourse are much lower in PSID-TAS than in NSFG or AFHS. Again, it is possible that the context of prior questions on lifetime experiences with all types of sexual relationships and a range of reproductive health issues improves reporting in NSFG/AFHS. Second, similar to the wider age range, men report forced intercourse at much lower rates than women. Third, in contrast to women, there is no evidence of a pandemic-Specific increase in experiences of forced intercourse among younger men.

### College Attendance and Forced Intercourse

For those aged 24–28, the 2011–2013 NSFG data indicate that women who either never attended college or who attended less than four years of college were *twice as likely* to have ever experienced forced intercourse compared to women who attended four or more years of college (p<0.01, Table 3). This result replicates findings reported previously (12). However, beginning with the 2015–2017 NSFG, this difference shrinks to become non-significant. The shrinking size of the difference in 2015–2017 is as much because of an increase in the rates of forced intercourse among women who had completed four or more years of college as it is because of a decline among women who had completed less than four years of college. The difference by level of college education continues to be non-significant when considering the PSID-TAS data (Table 3).

However, when considering the pandemic-Specific 2020–2022 AFHS data, the difference by years of college returns to more than double and significant, despite the smaller sample size (p<0.05, Table 3). The rates of forced intercourse among women aged 24–28 who completed four or more years of college during the pandemic are quite similar to those documented before the pandemic in the NSFG 2015–2017 data. But the rates of forced intercourse among women with less education during the pandemic are nearly double those in the NSFG 2015–2017. Not only did rates of forced intercourse among young women rise during the pandemic, the increase is mainly among women with less education.

## Discussion

Forced intercourse is a high-prevalence public health issue, a large-scale social issue, a safety priority, and a national concern. By 2017 the #MeToo movement increased popular attention to this issue, and may have reduced the stigma of reporting these experiences. The experience of forced intercourse has particularly high potential for long-term adverse health consequences (10). Even though forced intercourse represents less than half of all sexual assaults, our analyses reveal that about 1 in 5 U.S. women aged 18–49 have experienced forced intercourse. This total population rate of forced intercourse remained stable from 2011 to 2022. Given the substantial health and wellbeing risks associated with this experience, this high rate of forced intercourse continues to be a substantial national health risk.

The steps taken to control the spread of COVID-19 created circumstances that could have contributed to higher rates of forced intercourse. Although social distancing reduced entry into sexual relationships, for those already in sexual relationships rates of exit may have also been reduced. Opportunities for socializing declined, giving those in adverse intimate relationships less contact with others and potentially less opportunity to move. Rates of abuse and violence may have increased in adverse sexual relationships. Our results show that rates of forced intercourse among younger women—aged 24–28—increased in reports collected from late 2020 though early 2022 relative to previous reports.

The well-documented educational difference in forced intercourse shows that those who completed *less than* four years of college had *significantly higher rates* of forced intercourse than peers who completed four years of college (12). We found that this difference between those who completed four or more years of college and those who did not was declining before the pandemic. However, during the pandemic we found that those with less education again became *more than twice as likely* to experience forced intercourse.

Nationwide, the high levels of forced intercourse are likely to contribute to continued high levels of unintended pregnancies. Forced intercourse rarely involves effective contraception, increasing the probability of unintended pregnancy (14). Research on the long-term consequences of unintended pregnancies demonstrate adverse consequences for both the children and their siblings (31–34). Thus, public health interventions to reduce forced intercourse have the potential to improve both the health and wellbeing of victims and their families.

## Conclusion

Overall rates of forced intercourse have remained high in the U.S. throughout the past decade. Forced intercourse is a pervasive issue throughout the U.S. regardless of educational experience. However, nationally-representative measures collected during the pandemic (2020–2022) indicate that lifetime experience of forced intercourse significantly increased among females aged 24–28. Differences by college attainment also increased, with more than 30% of females aged 24–28 who had completed fewer than 4 years of college reporting ever being forced to have intercourse.

## Figures and Tables

**Table 1.** Percentage of the U.S. Population Aged 18–44/49 Reporting “Ever Forced to Have Intercourse”

Female					Male			
	NSFG 2011–2013	NSFG 2013–2015	NSFG 2015–2017	AFHS 2020–2022	NSFG 2011–2013	NSFG 2013–2015	NSFG 2015–2017	AFHS 2020–2022
Age	Percent (SE)^1^	Percent (SE)	Percent (SE)	Percent (SE)	Percent (SE)	Percent (SE)	Percent (SE)	Percent (SE)
18–19	15.1 (2.7)	10.6 (1.8)	16.1 (3.5)	3.7 (2.6)	3.9 (1.0)	2.7 (0.8)	4.6 (1.3)	8.7 (4.5)
20–24	15.0 (1.8)	18.8 (1.8)	15.8 (2.3)	24.3 (4.3)*	5.2 (0.9)	4.2 (0.8)	6.7 (1.6)	4.4 (2.2)
25–29	17.9 (1.8)	18.9 (1.8)	19.4 (2.0)	23.3 (3.9)	3.4 (0.8)	7.2 (0.9)	5.5 (1.1)	8.1 (3.0)
30–34	20.0 (1.9)	15.1 (1.4)	19.9 (2.4)	20.4 (3.4)	7.2 (1.4)	6.1 (1.0)	4.7 (1.1)	7.6 (2.8)
35–39	20.3 (2.0)	22.0 (2.0)	17.2 (1.9)	20.4 (3.5)	7.8 (1.5)	6.0 (1.3)	7.7 (1.3)	5.6 (2.2)
40–44	25.6 (2.2)	19.6 (1.5)	20.5 (2.4)	27.4 (4.7)	7.8 (1.6)	6.0 (1.3)	7.3 (1.7)	2.2 (1.0)
45–49			20.9 (2.5)	25.6 (5.1)			5.4 (1.5)	11.7 (4.3)
**Total**	**19.5 (0.8)**	**18.2 (0.7)**	**18.8 (0.9)**	**22.0 (1.7)**	**6.0 (0.6)**	**5.7 (0.5)**	**6.1 (0.5)**	**6.5 (1.1)**
n	4962	5054	4945	1042	4130	3861	3996	872

1 Linearized standard errors (SE) are in parentheses.

* A statistically significant increase in rates of forced intercourse.

**Table 2.** Percentage of the U.S. Population Aged 18–28 Reporting “Ever Forced to Have Intercourse”

		Female						Male				
	NSFG 2011–2013	NSFG 2013–2015	NSFG 2015–2017	PSID-TAS 2017	PSID-TAS 2019	AFHS 2020–2022	NSFG 2011–2013	NSFG 2013–2015	NSFG 2015–2017	PSID-TAS 2017	PSID-TAS 2019	AFHS 2020–2022
Age	Percent (SE)^1^	Percent (SE)	Percent (SE)	Percent (SE)	Percent (SE)	Percent (SE)	Percent (SE)	Percent (SE)	Percent (SE)	Percent (SE)	Percent (SE)	Percent (SE)
18–23	15.1 (1.7)	15.5 (1.2)	16.5 (2.1)	8.9 (1.4)	7.3 (1.3)	14.5 (3.1)	4.4 (0.7)	3.7 (0.7)	6.5 (1.3)	3.7 (1.0)	3.7 (1.1)	6.4 (2.4)
24–28	16.5 (1.7)	19.8 (1.6)	16.6 (2.1)	12.7 (1.9)	6.4 (1.4)	26.7* (3.9)	4.0 (0.8)	6.4 (1.0)	5.1 (1.0)	1.2 (0.5)	1.5 (0.6)	5.3 (2.2)
**Total**	**15.7** **(1.1)**	**17.5** **(0.9)**	**16.5 (1.4)**	**10.6** **(1.2)**	**6.9 (0.9)**	**19.9** **(2.5)**	**4.3 (0.5)**	**5.0 (0.6)**	**5.8 (0.9)**	**2.7** **(0.7)**	**2.8** **(0.7)**	**6.0 (1.7)**
n	2218	2187	1849	1311	1348	335	1944	1730	1554	1202	1220	261

1 Linearized standard errors (SE) are in parentheses.

* A statistically significant increase in rates of forced intercourse.

**Table 3.** Percentage of U.S. Females Aged 24–28 Reporting “Ever Forced to Have Intercourse” by Education

	NSFG 2011–2013		NSFG 2013–2015		NSFG 2015–2017		PSID-TAS 2017		PSID-TAS 2019		AFHS 2020–2022	
Educational attainment	Percent (SE)^1^	N	Percent (SE)	N	Percent (SE)	N	Percent (SE)	N	Percent (SE)	N	Percent (SE)	N
Less than 4 years of college	19.7 (2.3)	743	24.4 (2.1)	777	17.9 (2.3)	652	14.5 (2.5)	401	6.7 (1.7)	384	32.6 (5.7)	79
4 or more years of college	10.8 (1.9)*	289	10.8 (2.1)*	299	14.5 (3.1)	262	9.6 (3.0)	168	4.3 (1.8)	168	16.4 (4.0)*	102
**Total**	**16.5 (1.7)**	**1,032**	**19.8 (1.6)**	**1,076**	**16.6 (2.1)**	**914**	**12.7 (1.9)**	**569**	**5.8 (1.2)**	**552**	**26.7 (3.9)**	**181**

1 Linearized standard errors (SE) are in parentheses.

* A significant difference in rates of forced intercourse between those who had completed 4 or more years of college and those who did not.

## Data Availability

This research uses publicly available data from the U.S. National Survey of Family Growth (2011–2017 public releases; https://www.cdc.gov/nchs/nsfg/index.htm) and the U.S. Panel Study of Income Dynamics – Transition into Adulthood Supplement (2017 and 2019 wave public releases; https://psidonline.isr.umich.edu/). Data from the American Family Health Study (AFHS) is being processed for public release (https://afhs.isr.umich.edu/).

## Abbreviations

AFHS: American Family Health Study
ACASI: Audio-Computer Assisted Self-Interviewing
NSFG: National Survey of Family Growth
NCHS: U.S. National Center for Health Statistics
NCVS: U.S. National Crime Victimization Survey
PSID-TAS: U.S. Panel Study of Income Dynamics Transition into Adulthood Supplement
